# Is being a 'left-behind' child associated with an increased risk of self-poisoning in adulthood? Findings from a case–control study in Sri Lanka

**DOI:** 10.1136/bmjgh-2020-003734

**Published:** 2021-03-01

**Authors:** Duleeka Knipe, Paul Moran, Laura D Howe, Piumee Bandara, Kolitha Wickramage, David Gunnell, Thilini Rajapakse

**Affiliations:** 1 Population Health Sciences, University of Bristol, Bristol, UK; 2 South Asian Clinical Toxicology Research Collaboration, Faculty of Medicine, University of Peradeniya, Peradeniya, Sri Lanka; 3 Centre for Academic Mental Health, Bristol Medical School, University of Bristol, Bristol, UK; 4 MRC Integrative Epidemiology Unit, University of Bristol, Bristol, Bristol, UK; 5 Translational Health Research Institute, Western Sydney University, Penrith South, New South Wales, Australia; 6 International Organization for Migration, Geneve, Switzerland; 7 National Institute of Health Research Biomedical Research Centre, University Hospitals Bristol NHS Foundation Trust, Bristol, UK; 8 Department of Psychiatry, Facultly of Medicine, University of Peradeniya, Peradeniya, Central, Sri Lanka

**Keywords:** suicide, child health, mental health & psychiatry, epidemiology, poisoning

## Abstract

**Purpose:**

The long-term consequences of parental emigration on offspring self-harm risk is unknown.

**Methods:**

We investigated the association between experiencing parental emigration in childhood with hospital presentations for self-poisoning in adulthood using a hospital case–control study. Cases were adult self-poisoning patients (≥18 year olds) admitted to the medical toxicology ward Teaching Hospital Peradeniya, Sri Lanka. Sex and age frequency matched controls were recruited from the outpatient department or nearby specialist clinics at the same hospital. Details of parental emigration were collected using a pre-piloted questionnaire. The relationship between parental emigration and self-poisoning in adulthood was estimated using logistic regression models.

**Results:**

298 cases, and 500 hospital controls were interviewed for the study. We estimate that one in five adults experienced parental emmigration as children (95% CI 17% to 24%). We find limited evidence that children from households with emigrating parents were more likely to experience adverse childhood experiences than those with non-emigrating parents. We found no statistical evidence of an increased risk of self-poisoning in adulthood in individuals who experienced parental emigration (maternal or paternal) during childhood. There was no statistical evidence that the impact differed by the sex of the participant.

**Conclusion:**

Adults who experienced parental emigration as children were no more likely to self-poison than adults with non-emigrating parents. Further research using longitudinal data are needed to understand whether any adverse outcomes observed in 'left-behind' children are a consequence of parental emigration or due to factors associated but predate the emigration. Prospective data are also important to investigate whether there are any lasting effects on children who experience parental emigration.

**Key questions**

**What is already known?**

- The emigration of parents from low-income and middle-income countries is a growing phenomenon and there are concerns over the children 'left behind' in countries of origin.
- Amidst these concerns strict and discriminatory migration policies are being introduced which restrict the emigration of mothers.
- The long-term health consequences (ie, into adulthood) of this parental emigration is unknown.

**What are the new findings?**

- Experiencing parental emigration during childhood (≤18 years) was not associated with an increased risk of suicidal behaviour in adulthood in Sri Lanka.

**What do the new findings imply?**

- The long-term benefits of parental emigration may outweigh any short-term negative mental health/distress consequences.
- There is likely to be a complex interplay of a variety of factors which exist before, during and after the migratory cycle.
- This study only looked at one outcome (self-poisoning) in one context—the lack of research into the long-term consequences of parental emigration needs to be further explored to support healthy migration.

## Introduction

There are approximately 272 million international migrants globally, with 40% of these migrants originating from Asia, especially South Asia.^1^ Much of this migration is for labour within Asia, and in some Asian countries, remittances provide an important contribution to the overall economy (eg, Philippines, Sri Lanka). The migration of low-waged workers within Asia often occurs on short-term contracts, negating the possibility of family members being able to migrate with the labour worker. This results in families being dispersed across countries, with parents separated from their children.^2^ The number of affected children is unclear, but it is thought to be in the hundreds of millions.^3^

There is growing concern over the well-being of children 'left behind' as a consequence of parental labour migration. The term 'left-behind' has been used to describe children who have experinced parental migration (internal or external). We have avoided this term as it implies adverse trajectories, and fits with the UN migration agency's position to not describe children of migrant workers as being 'left-behind'. There is potentially a trade-off between increased family income, resultant improved educational opportunities for children and parental absence and the potential adverse consequences of this. Parental absence during development can affect future emotional resilience and may also lead to reduced supervision of the child. However, it could be argued that in many Asian countries (like Sri Lanka) where extended families are common,^4^ the absence of a parent may be felt less acutely by the child, as some of the negative impacts may be mitigated by the presence of other family members. We know relatively little about whether individuals who experience parental emigration during childhood experience increased adverse consequences or have improved educational outcomes.

The current evidence on the impact of parental migration (without their children) on offspring has largely focussed on short-term outcomes, often during the time of disruption compared with children with more stable family environments.^3^ It is important to remember that any disruption to the family environment will result in some psychological distress in children. A recent meta-analysis indicated that parental migration is associated with poor mental and physical health outcomes in their offspring.^3^ The findings of this review, however, indicated that our understanding regarding the impact of parental emigration (ie, across national borders) is limited—only 21 studies investigated international migration, with only 10 studies exploring offspring mental health. Furthermore, none of the studies included in this review looked at self-harm, or the long-term (ie, into adulthood) mental health consequences of parental migration on children who stayed at home.

Using data collected from a large case–control study in Sri Lanka, we aimed to answer the following questions: (1) are there sociodemographic and clinical (ie, depression and alcohol use disorders) differences between individuals who experience parental emigration versus those who do not?; (2) is parental emigration associated with an increased risk of self-poisoning (the most common method of non-fatal self-harm in Sri Lanka) in adulthood?; (3) is there evidence that any associations vary by the sex of the child? and (4) are there certain migration characteristics (age of first parental emigration, frequency of emigration, unskilled/skilled emigration, frequency of contact and who remittances were sent to) which are associated with an increased risk of self-poisoning?

## Methods

This study uses data collected as part of a large hospital-based case–control study investigating the association between adverse childhood experiences and self-poisoning risk in adulthood.^5^ The main study findings related to adverse childhood experiences is presented elsewhere.^6^ The current study focusses on the impact of parental emigration, an experience that cannot be clearly classed as an adverse experience given the potential beneficial impacts related to this exposure and therefore is investigated separately to other known adverse exposures. Data were collected from the Teaching Hospital Peradeniya (THP) in the Kandy district in the Central Province of Sri Lanka. Most people (81%) live in rural areas, with a Buddhist and Sinhala majority.^7^ The catchment area of the THP has a large Tamil population—a minority ethnic group who are more likely to experience poverty.^8^ Roughly 20 000 people emigrate (65% for low-wage work) from the Kandy district (ie, 1.4% of the population emigrate each year).^9^ The Kandy district is the third largest district for labour emigration. Sri Lanka has a free government healthcare system, and hospitals with major medical subspecialities are distributed through all districts of the country.^10^ The prevalence of common mental disorders and incidence of suicide in Kandy district is consistent with the national average^11 12^ (excluding the post-conflict areas which tend to have higher rates^13^).

### Patient and public involvement

The research question and study design were informed by discussions with community members and full details are provided in our published protocol,^5^ and described here in brief. A series of patient and public involvement (PPI) workshops were conducted, engaging a range of stakeholders both at the local community and government ministry level. One of the workshops was with returnee migrant workers and family members of current migrants. These workshops explored possible pathways to suicidal behaviour. Child abuse, maltreatment and neglect were highlighted as important risk factors for suicidal behaviour. Many members of the PPI workshops highlighted that a key perceived issue affecting the development and well-being of children was the emigration of parents, particularly mothers. The information gathered from these discussions were used to inform the design of the questionnaire and analysis.

### Cases

Cases were adult self-poisoning patients (≥18 years old) admitted to the medical toxicology ward between July and December 2018. All poisoning presentations at THP are admitted to the toxicology ward for treatment. Self-poisoning cases were identified through the ward admission book and verbally confirmed through patient self-report. Only patients with self-poisoning (regardless of suicidal intent but excluding accidental poisoning) were included in this study, and we did not recruit people who had self-harmed using other methods; however self-poisoning is the most common method of non-fatal self-harm (99%) in Sri Lanka.^14^

### Controls

Sex and age (±5 years age strata) frequency matched controls were recruited from individuals (both patients and accompanying visitors) presenting to the THP outpatient department (OPD) or nearby specialist clinics. We did not perform individual matching of cases and controls (although we kept a rough record of this for recruitment purposes), but chose to match on age and broad age groups to allow for a similar age and sex distribution among cases and controls. Individuals waiting to be seen by a doctor or waiting for someone to be seen (ie, accompanying visitor) were approached to be interviewed. Interviews were only conducted if the individual was able to accompany the data collector to a designated confidential space nearby and to give adequate time for interview. We aimed to recruit 1 control for each case and aimed to recruit 200 cases and controls. This was to allow us to be able to detect a twofold difference in risk with 86% power (alpha=0.05) for a childhood exposure that was reported in 20% of the control population. In the absence of a primary care infrastructure, the OPD provides treatment for conditions which would normally be seen in this setting (eg, coughs, hypertension). Individuals who reported a self-harm episode in the past were excluded from the study, and additional controls were recruited. The original protocol intended to recruit 200 cases and 200 controls over a 6-month period assuming a self-poisoning admission rate of 100 cases per month. During the first 2 months of the study we noted that the admission rate was one-third of the assumed rate. We also noted that our response rate for controls was lower than we originally anticipated. The study team, therefore, decided to increase control recruitment (at least two controls per case) to ensure the study was adequately powered—on average we recruited two controls for each case.

In addition to a hospital control series, we also collected community controls from the local population from the main catchment areas for THP and where most cases resided. We did this in order to examine (in a sensitivity analysis) the effect of potential selection bias that might have been introduced by the use of hospital controls. A random sample (n=12 out of 159) of villages (in two administrative divisions of Kandy District which were identified as being the main catchment area for THP) were identified and age and sex frequency matched controls were recruited door-to-door. For every household approached, only one participant matched on sex and age (±5 years) was selected for interview. If more than one participant was eligible, the participant with the most recent birthday was selected for an interview. We attempted to recruit two controls for every case. For resource and logistical reasons (ie, topography of the region) not all households in the village could be reached. The community controls were recruited after the hospital cases and controls (19 January 2019 and 2 April 2019) and were not part of the original protocol.^5^

Cases and controls who were physically unable or too unwell to participate, and those identified as being cognitively impaired, were excluded from the study (see online supplemental figure 1).

10.1136/bmjgh-2020-003734.supp1 Supplementary data

### Data collection

A face-to-face interview was conducted with each participant. All interviews were conducted by trained interviewers in the preferred local language of the participant in a private setting. Interviewers were given a standard script to follow and were regularly shadowed by the study supervisor to ensure a consistent approach was adopted.

The main exposure (parental emigration) was collected via a series of pre-piloted questions related to maternal and paternal economic emigration. Details of the emigration were also collected (frequency of migration; age of participant at each migratory episode; parental contact during the migration; type of occupation parent migrated for (skilled/unskilled); and data on who remittances were sent to).

Sociodemographic data (ethnicity, religion, educational level and parental education) were collected via a pre-piloted questionnaire. Adverse childhood experiences were measured using the WHO’s Childhood Adversity Scale^15^ and presented as a separate analysis. The scale was translated to the local languages, piloted and adapted for local use prior to the study. We used data on family factors, relevant during the first 18 years of life, collected as part of this scale for this study. The locally validated Patient Health Questionnaire-9 (PHQ-9)^16^ and Alcohol Use Disorders Identification Test (AUDIT)^17^ were used to measure depression and alcohol use disorders (respectively).

### Statistical analysis

We used the hospital control data to describe the sociodemographic, household and clinical differences between those who experienced parental emigration versus those who did not. A score of 10 or more on the PHQ-9 was used to indicate moderate-to-severe depression,^16^ and AUDIT scores of 8 or more indicated hazardous drinking behaviour. The primary inferential analysis included data from cases and hospital controls with complete data on exposure to parental emigration, sociodemographic information and the specific childhood adversity questions used in this analysis.

The relationship between parental emigration and self-poisoning in adulthood was estimated using a series of unconditional logistic regression models. This differs from the original protocol where we specified a matched analysis (ie, conditional logistic regression). We deviated from the protocol to increase the statistical precision of the study without losing validity.^18^ Given that the study recruited frequency age-matched and sex-matched controls, all models were adjusted for age and sex of the participant. The main model adjusted for age, sex, ethnicity, religion and childhood socioeconomic position (highest level of parental education). We did not include any adverse childhood experiences (eg, violence in the home) as covariates in our regression models given that these experiences may lie on the causal pathway, and it is not possible to disentangle whether they predate the emigration, or lie between emigration and self-poisoning behaviour. We also stratified our analysis by sex and formally tested whether this modified any associations we observed (test for interaction). In addition, given the concerns over maternal emigration, we also estimated the risk of self-poisoning in adulthood based on the sex of the parent who emigrated (categorical exposure variable: no emigrating parent; maternal emigration; paternal emigration; maternal and paternal emigration).

Our secondary analysis explored whether certain characteristics associated with the parent’s emigration (frequency of migration; age of child at first migratory episode; parental contact during the migration; type of occupation parent migrated for (skilled/unskilled); and data on who remittances were sent to) altered any associations observed by adding them as exposure variables into our models. We did this for each exposure variable in turn. As a sensitivity analysis we explored the association between parental emigration and self-poisoning using the community control sample, to see whether the use of a different control group altered our conclusions. We also conducted a sensitivity analysis using all available data (ie, including all cases and controls regardless of whether they had missing covariate data). Stata V.16 was used for all analysis.^19^

## Results

A total of 298 (87% response rate) cases, 500 (62%) hospital controls and 455 (63%) community controls were interviewed for the study (online supplemental figure 1). While there were no sex differences between cases who consented to taking part in the survey, more female than male hospital controls responded. We only have data on age for non-responding cases, and they tended to be older (median age: responders 26 years; non-responders 33 years). Cases had a higher degree of missing data (complete data n=239, 80%) than hospital controls (n=456, 91%). However, most variables had a low degree of missing data (≤1%) except for parental education (12%).

There were more female (57%) than male (44%) participants, with a median age of 25 years (interquartile interval (IQI) 20–34) for cases and 26 years (IQI 21–36) for hospital controls (table 1). A higher proportion of cases (compared with hospital controls) were non-Sinhala (cases vs controls: 21% vs 8%), non-Buddhist (24% vs 10%) and had a lower childhood socioeconomic position (parents with no schooling: 7% vs 2%). Cases were also more likely to report living with someone with a mental health issue, were more likely to have experienced parental separation/divorce/death and violence in the home in the first 18 years of life.

**Table 1:** Characteristics of cases and hospital controls for key variables

	N (%)			P value*
	Cases	Hospital controls	Community controls	
No. of complete cases (% complete)	239 (80.2)	456 (91.2)	410 (90.1)	
Male n (%)	104 (43.5)	182 (39.9)	166 (40.5)	0.07
Age median (IQI)	26 (32–36)	26 (21–37)	28 (22–39)	0.39
Ethnicity				
Sinhala	189 (79.1)	419 (91.9)	358 (87.3)	<0.001
Tamil	30 (12.6)	16 (3.5)	34 (8.3)	
Moor/Burgher	20 (8.4)	21 (4.6)	18 (4.4)	
Religion				
Buddhist	182 (76.2)	412 (90.4)	347 (84.6)	<0.001
Non-Buddhist	57 (23.8)	44 (9.6)	63 (15.4)	
Parent’s highest education				
No schooling	17 (7.1)	7 (1.5)	6 (1.5)	<0.001
Primary	105 (43.9)	150 (32.9)	139 (33.9)	
Passed O-Level	56 (23.4)	139 (30.5)	116 (28.3)	
Passed A-Level	61 (25.5)	160 (35.1)	149 (36.3)	
Family factors				
Alcohol/drug misuse‡†	48 (20.1)	85 (18.6)	80 (19.5)	0.65
Mental health issues/suicidal§	35 (14.6)	37 (8.1)	36 (8.8)	0.01
Jail/prison¶	20 (8.4)	27 (5.9)	20 (4.9)	0.22
Parental separation/divorce	27 (11.3)	15 (3.3)	19 (4.6)	<0.001
Parental death	36 (15.1)	31 (6.8)	47 (11.5)	<0.001
Violence in the home**	48 (20.1)	49 (10.7)	58 (14.1)	0.001

*χ^2^ and Mann-Whitney U tests comparing cases with hospital controls.

†Questions from the WHO adverse childhood experiences questionnaire. In the first 18 years of life: *Did you live with a household member who was a problem drinker or alcoholic, or misused street or prescription drugs?

‡Did you live with a household member who was depressed, mentally ill or suicidal?

§Did you live with a household member who was ever sent to jail or prison?

¶Defined as having witnessed (seen or heard) psychological (many times) or physical (few/many times) abuse of a parent or other household member.

IQI, interquartile interval.

Table 2 presents the sociodemographic characteristics of participants from the hospital controls who experienced parental emigration in childhood versus those who did not. One in five hospital controls experienced parental emigration in childhood (20%, 95% CI 17% to 24%), and 22% (95% CI 18% to 26%) of community controls. A higher proportion of non-Sinhala (Tamil and Moor), non-Buddhist individuals reported parental emigration. There were no differences in the educational attainment of adults who experienced parental emigration during childhood. Adults who reported parental emigration were exposed to certain adverse family experiences to the same degree as those who did not experience parental emigration. The only exceptions were that parental emigration was associated with a lower level of parental death in hospital controls, and increased household violence (although there was only weak statistical evidence of this association in hospital controls, with stronger evidence in community controls). Community controls who experienced parental emigration in childhood also reported parental separation/divorce and living with someone who was imprisoned than those who did not experience parental emigration. In addition, adults who experienced parental emigration in childhood in the hospital controls sample were just as likely to screen positive for depression (16%) and alcohol use disorders (12%) as those who did not experience parental emigration (depression: 16%; alcohol use disorders: 11%). This was also consistent with the findings in the community control series.

**Table 2:** Characteristics of individuals who experienced parental migration versus those who did not in the hospital and community control series

	Hospital controls			Community controls		
	Parental migration N (%)			Parental migration N (%)		
	No	Yes	P value	No	Yes	P value
n=	364	92		321	89	
Male n (%)	145 (39.8)	37 (40.2)	0.95	129 (40.2)	37 (41.6)	0.81
Ethnicity						
Sinhala	340 (93.4)	79 (85.9)	0.03	287 (89.4)	71 (79.8)	0.05
Tamil	12 (3.3)	4 (4.3)		23 (7.2)	11 (12.4)	
Moor/Burgher	12 (3.3)	9 (9.8)		11 (3.4)	7 (7.9)	
Religion						
Buddhist	336 (92.3)	76 (82.6)	0.01	281 (87.5)	66 (74.2)	0.002
Non-Buddhist	28 (7.7)	16 (17.4)		40 (12.5)	23 (25.8)	
Education level						
No schooling	2 (0.5)	0 (0)	0.29	0 (0)	0 (0)	0.33
Completed grades 1–10	68 (18.7)	17 (18.5)		60 (18.7)	23 (25.8)	
Passed O-Level	93 (25.5)	32 (34.8)		91 (28.3)	23 (25.8)	
Passed A-Level	201 (55.2)	43 (46.7)		170 (53.0)	43 (48.3)	
Parent’s highest education						
No schooling	7 (1.9)	0 (0)	0.21	5 (1.6)	1 (1.1)	0.74
Completed grades 1–10	122 (33.5)	28 (30.4)		112 (34.9)	27 (30.3)	
Passed O-Level	104 (28.6)	35 (38.0)		87 (27.1)	29 (32.6)	
Passed A-Level	131 (36)	29 (31.5)		117 (36.4)	32 (36.0)	
Family factors						
Alcohol/drug misuse*	69 (19)	16 (17.4)	0.73	59 (18.4)	21 (23.6)	0.27
Mental health issues/suicidal†	30 (8.2)	7 (7.6)	0.84	28 (8.7)	8 (9.0)	0.94
Jail/prison‡	21 (5.8)	6 (6.5)	0.79	11 (3.4)	9 (10.1)	0.01
Parental separation/divorce	11 (3)	4 (4.3)	0.52	11 (3.4)	8 (9.0)	0.03
Parental death	30 (8.2)	1 (1.1)	0.02	33 (10.3)	14 (15.7)	0.15
Violence in the home§	34 (9.3)	15 (16.3)	0.05	38 (11.8)	20 (22.5)	0.01
Moderate/severe depression¶	59 (16.2)	15 (16.3)	0.98	34 (10.6)	12 (13.5)	0.44
Hazardous drinking**	40 (11.0)	11 (12.0)	0.79	29 (9.0)	14 (15.7)	0.07

*Questions from the WHO adverse childhood experiences questionnaire. In the first 18 years of life: *Did you live with a household member who was a problem drinker or alcoholic, or misused street or prescription drugs?

†Did you live with a household member who was depressed, mentally ill or suicidal?

‡Did you live with a household member who was ever sent to jail or prison?

§Defined as having witnessed (seen or heard) psychological (many times) or physical (few/many times) abuse of a parent or other household member.

¶Based on a Patient Health Questionnaire (PHQ-9) score of 10 or more.

**Based on an Alcohol Use Disorders Identification Test (AUDIT) of 8 or more.

We found no statistical evidence of an association between parental emigration in childhood and self-poisoning behaviour in adulthood (Model 2, table 3). We did not observe any differences in the risk of self-poisoning in adulthood based on the sex of the parent who emigrated (the 95% CIs overlap for the risk associated with maternal vs paternal emigration). In the sex-stratified analysis, there was an indication that the risk of self-poisoning in adulthood was higher in women who experienced parental emigration (OR 1.69, 95% CI 1.02 to 2.79). There was, however, no statistical evidence that the association between parental emigration and self-poisoning was modified by the sex of the left-behind child (p value for interaction 0.16).

**Table 3:** Adjusted and sex-stratified associations of parental emigration and hospital presentation for self-poisoning in adulthood

		N (%)			
		Cases	Controls	Model 1	Model 2
Overall	Parental migration				
	No	178 (74.5)	364 (79.8)	1	1
	Yes	61 (25.5)	92 (20.2)	1.30 (0.89 to 1.90)	1.28 (0.87 to 1.90)
	Which parent migrated?				
	No migration	178 (74.5)	364 (79.8)	1	1
	Maternal	36 (15.1)	51 (11.2)	1.40 (0.88 to 2.23)	1.26 (0.77 to 2.05)
	Paternal	21 (8.8)	30 (6.6)	1.37 (0.76 to 2.47)	1.49 (0.80 to 2.74)
	Both	4 (1.7)	11 (2.4)	0.69 (0.22 to 2.22)	0.82 (0.25 to 2.71)
Males	Parental migration*				
	No	83 (79.8)	145 (79.7)	1	1
	Yes	21 (20.2)	37 (20.3)	0.98 (0.53 to 1.80)	0.90 (0.47 to 1.73)
Females	Parental migration*				
	No	95 (70.4)	219 (79.9)	1	1
	Yes	40 (29.6)	55 (20.1)	1.55 (0.96 to 2.51)	1.69 (1.02 to 2.79)

Model 1 – adjusted for age and sex.

Model 2 – adjusted for age, sex, ethnicity, religion, childhood socioeconomic position.

*P value for interaction (Model 2): p=0.16.

As a secondary analysis we explored whether certain migratory characteristics (frequency of migration; age of child at first migratory episode; parental contact during the migration; type of occupation parent migrated for (skilled/unskilled); and who remittances were sent to) of the parent’s emigration were associated to a greater or lesser degree with risk of self-poisoning (table 4) but found no evidence that this was the case. Fewer cases and controls were included in this analysis due to a higher degree of missing data in the migration specific data variables. Our sensitivity analysis using community controls was consistent with the primary analysis, although we did not observe any statistical evidence of an increased risk of adulthood self-poisoning in female children left behind (online supplemental table 1). The analysis using all available data (ie, including participants with missing data for some of the variables) were consistent with the main results (online supplemental table 2).

**Table 4:** Distribution and confounder adjusted associations of characteristics of parental emigration and hospital presentation for self-poisoning in adulthood

	N(%)		OR 95% CI*
	Cases	Controls	
No. of complete cases (% complete)	214 (71.8)	421 (84.2)	
Age at first migration (years)			
No migration	178 (83.2)	364 (86.5)	1
≤5	14 (6.5)	23 (5.5)	1.15 (0.56 to 2.37)
>5	22 (10.3)	34 (8.1)	1.16 (0.64 to 2.11)
Number of times migrated			
No migration			1
1	16 (44.4)	28 (49.1)	1.09 (0.56 to 2.14)
>1	20 (55.6)	29 (50.9)	1.21 (0.64 to 2.28)
Type of migration			
No migration	178 (83.2)	364 (86.5)	1
Low-waged	31 (14.5)	43 (10.2)	1.31 (0.77 to 2.22)
non-low waged/mixed	5 (2.3)	14 (3.3)	0.69 (0.23 to 2.05)
Remittances sent back to			
No migration	178 (83.2)	364 (86.5)	1
Father	32 (15.0)	54 (12.8)	1.07 (0.65 to 1.78)
Mixture of recipients	4 (1.9)	3 (0.7)	2.57 (0.55 to 12.11)
Contact with migrant parent			
No migration	178 (83.2)	364 (86.5)	1
Never/<once a month	10 (4.7)	12 (2.9)	1.62 (0.66 to 3.98)
More than once a month	26 (12.1)	45 (10.7)	1.03 (0.60 to 1.79)

*Adjusted for age, sex, ethnicity, religion, childhood socioeconomic position. Each exposure variable (emigration characteristic) is modelled separately.

## Discussion

To the best our knowledge, this is the first study to investigate the long-term mental health consequences of parental emigration on children 'left behind' in South Asia. Among both our control groups (hospital and community), one in five adults experienced parental emigration as children. We find no statistical evidence of an increased risk of self-poisoning in adulthood in individuals who experienced parental emigration during childhood.

This study finds that 20% of adults in the Central Province of Sri Lanka experienced parental emigration as children. Comparative data are scarce, but this is consistent with unpublished pilot data collected by the authors in the North Central Province of Sri Lanka, and with another major domestic labour sending country—the Philippines (27%).^20^ In 1977, Sri Lanka became an open market economy and relaxed its restrictions on foreign migration, which led to an increase in low-waged migration.^21^ Children born during this time were, therefore, more likely to experience parental emgiration. Based on the 2019 mid-year population estimates for adults aged 20 to 44, we estimate that roughly 1.6 million (95% CI 1.4 to 1.9) adults experienced parental emigration during childhood.

Comparative studies investigating the long-term mental health consequences of parental migration (without their children) on offspring are scant. Some prior studies have investigated the association between current parental emigration and poor mental health but have reported inconsistent findings. Most studies find no statistical evidence of an association between current parental emigration and poor mental health (conduct disorder, depression, anxiety and suicidal thoughts).^22–27^ This is consistent with the evidence from the current study for parental emigration. One study from the Philippines reported a decreased risk of conduct disorders in children related to both maternal and paternal migration.^22^ Whereas evidence from Thailand, Romania, Trinidad and Tobago report higher levels of poor mental health in children.^22 28 29^ A study from Sri Lanka also found children who experience parental emigration were more likely to have higher psychopathology scores (unadjusted associations).^30^ There were no studies investigating the association between parental emigration and self-harm. There is some evidence in the Chinese literature which indicates that children currently 'left-behind' by parental migration within China have an increased odds of self-reported suicide attempts in the previous 12 months (unadjusted OR 2.7, 95% CI 1.3 to 5.9).^31^ The variability in the studies are likely to reflect contextual differences, as well as differences in the characteristics of the parents who migrate. For example, compared with the other countries listed above, the migrant parents in the Philippines are celebrated as ‘new heroes’, with strong government backed systems to support both the migrant and their families.^32^

In Sri Lanka migrant workers (especially female migrants) and their families do not have the same social standing in their community. These families are considered to be ‘dysfunctional’ and there is a preconception that if the parent migrates (especially the mother) the family will have poor outcomes.^33^ Local concern over the well-being of these 'left-behind' children led to the introduction of a draconian policy in 2013/2014 which restricted the migration of socioeconomically disadvantaged mothers with young children.^34^ The policy banned women with children under the age of 5 from migrating for domestic work (ie, low-waged labour) and required women with children over the age of 5 to complete a family background report (FBR). The completion of this FBR ‘ensures’ that the migrating mother has arranged alternative care for her children and that her husband or father approves her migration. In 2015 the policy was amended to include all female migration (not just those migrating for low-waged work). This population-level intervention was based on a weak evidence base, and is a major violation of the human rights of women with young children who wish to migrate.^35^ However, the concern over the well-being of children has meant that the policy has remained in place. Despite this concern, the evidence from this study indicates that there is no evidence that maternal (or paternal) emigration is associated with an elevated risk of self-poisoning in adulthood. The emigration of a parent may result in improved educational opportunities and standards of living for children who stay at home via increased family income, and/or changes to the home environment. The remittances sent back may also be used to improve the social standing and economic opportunities (ie, through investments for new income generating activities, eg, retail businesses).^36^ These may be possible explanations for the lack of any associations between parental emigration and self-poisoning in this study.

A further possibility is that the emigration of a parent may lead to family restructuring which results in a child being moved into a more stable home environment (eg, to live with grandparents or other relatives)—this may especially be the case if the mother is emigrating for reasons related to domestic abuse. In a culture where it is socially unacceptable for a woman to leave her husband, even in the context of domestic violence, emigration is often used as a socially sanctioned means of escape. Removing this form of escape (as is done by the current Sri Lankan policy), for these vulnerable women, is likely to lead to increased irregular migration (ie, informal unregulated labour migration) and increase the likelihood of exploitation.^33^ Trapping women in abusive relationships may lead to increases in self-harming behaviour in both the women and offspring in the household.^6 37–39^ In addition to domestic violence, poverty and spousal illness/substance abuse are also significant push factors for emigration. The current study finds evidence that adults who experienced parental emigration during childhood also reported experiencing violence in the home. The impact on the child, of parental (especially maternal) emigration, occurring against an already existing background of domestic violence and poverty, needs to be delineated further. Previous work has highlighted the important role of violence in the home, family disputes, alcohol misuse and issues related to gender norms (particularly in relation to the sexual property of young women—the group most likely to self-poison in this study) in contributing to self-poisoning risk in Sri Lanka.^38–42^ The nature of the care of migrant workers' offspring by extended family may also be an important mediating factor,^43^ especially as these children in Asia are often taken care of by their kin network, which may contribute to better well-being outcomes for children. In the context of these adverse conditions and complex interplay of mediating factors, prospective studies are urgently needed to determine whether any differences in offspring mental health observed in migrant verses non-migrant families pre-date or post-date the migratory episode.

It has been previously suggested that the impact of parental emigration (specifically maternal emigration) has a greater impact on female versus male children, due to female children taking on more domestic and childcare duties typically carried out by a mother,^26 44 45^ although the extent to which this occurs will differ by context. Despite this, few studies have formally explored in their analysis whether the sex of the child who experienced parental emigration alters any associations observed with mental health outcomes. Adhikari *et al* (2014) finds that female children who experince maternal emigration had a higher odds (OR 3.59 95% CI 1.50 to 8.62) of screening positive for poor mental health than male children (OR 2.00, 95% CI 0.89 to 4.52).^23^ However, they did not formally test for whether sex altered the association observed, and the CIs (ie, overlapping intervals) suggest that female children were no more likely to experience poor outcomes, than male children.^23^ In our sex-stratified analysis the point estimate for males who experienced parental emigration in childhood compared with those who were not, was close to the null (OR 0.90, 95% CI 0.47 to 1.73), while the estimate in women was indictive of increased risk (OR 1.69, 95% CI 1.02 to 2.79). We are, however, likely to be underpowered to be able to either confirm or rule out that the impact of parental emigration differed by the sex of the child.

This study has several strengths. To the best of our knowledge, this is the first exploration of the impact of parental separation as a consequence of parental emigration on adult suicidal behaviour (ie, long-term impacts). It uses data collected from a large case series and both hospital and community controls. The sensitivity analysis using community controls allowed us to address possible selection biasses that might have been introduced by using hospital controls—we observed consistent findings when using either control group. There are, however, methodological considerations to consider when interpreting the findings. We asked about past exposure to parental emigration, and it is possible that cases might have been more likely to recall parental absence than controls. We attempted to limit this recall bias by using a standard script for interviews. There was a higher degree of missing data in our case series than our control series which may have biassed our results. Despite asking questions about the migration history of parents, we were limited in our ability to answer important questions given the amount of missing data related to the migration specific questions—data collectors reported that this was due to difficulties in recalling these details. We also did not collect data on the duration of each migratory episode (ie, the length of the separation) and care provision during parental separation. Also, the study size means that we may not be able to rule out potentially important elevations in risk, as the study was only powered to identify a doubling in risk with 200 cases and 200 controls. Even though we increased the control sample, this increase will only have allowed us to detect an OR of 1.7 with 80% power (alpha=0.05) (post hoc power calculation performed using the *power* command in Stata V.16^19^).

This study adds important data to the current debate surrounding the well-being of children who experience parental migration. In settings like Sri Lanka where local opportunities for low-skilled work with reasonable pay are scarce, emigration provides an important gateway for families to escape poverty. The concern over the children who experience parental migration (particularly maternal migration) has led to severe emigration restrictions in Sri Lanka. This study finds limited evidence that children from households with a parent migrant are no more likely to experience adverse childhood experiences than those with non-migrant parents. This has important implications for policy and suggests that parental emigration and its impacts on children needs further attention.
