# Supplementary material for: Is being a 'left-behind' child associated with an increased risk of self-poisoning in adulthood? Findings from a case–control study in Sri Lanka
Source: BMJ Glob Health. 2021 Mar 1;6(3):e003734. doi: 10.1136/bmjgh-2020-003734 (PMC7925243; doi:10.1136/bmjgh-2020-003734)
Supplement: Supplementary data [file bmjgh-2020-003734supp001.pdf]

Supplementary material for manuscript: Is being a left-behind child associated with an increased risk of self-poisoning in adulthood? – findings from a case control study in Sri Lanka

Supplementary figure 1 – Participant recruitment for cases and controls

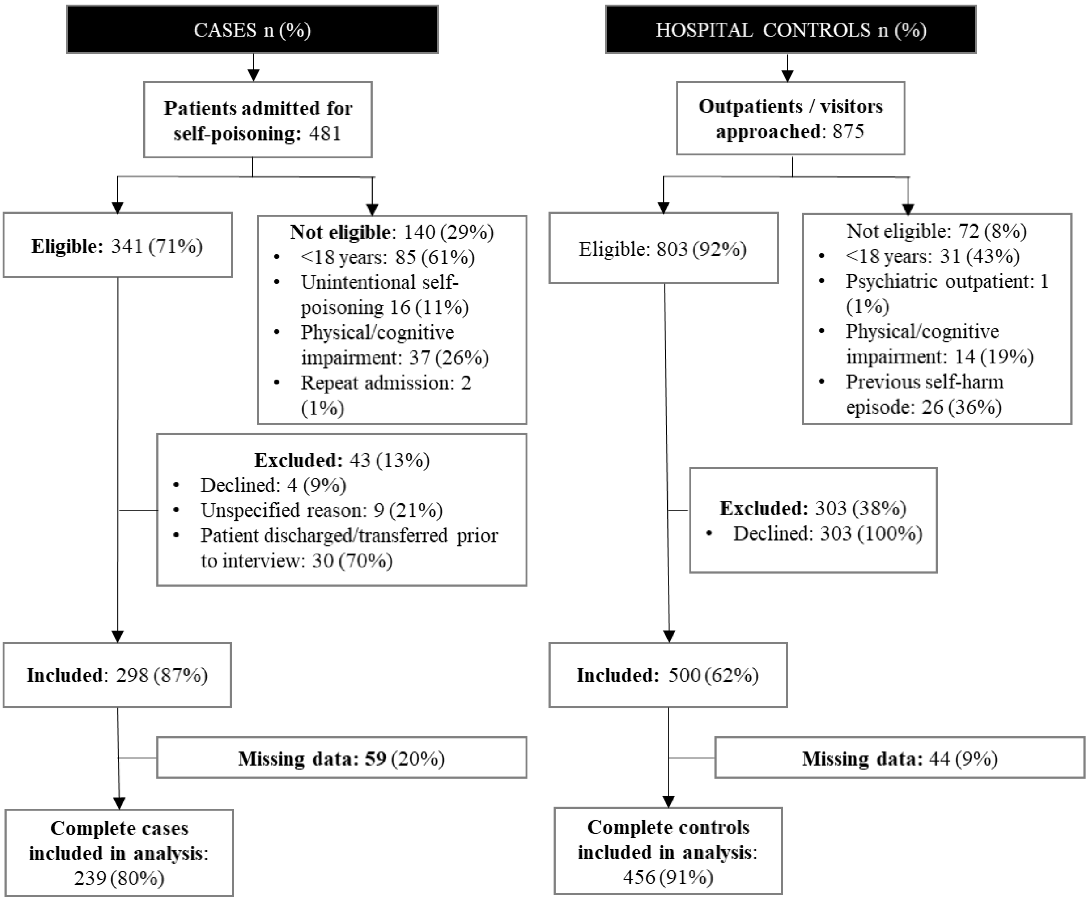

Knipe D, *et al. BMJ Global Health* 2021; 6:e003734. doi: 10.1136/bmjgh-2020-003734

Supplementary material for manuscript: Is being a left-behind child associated with an increased risk of self-poisoning in adulthood? – findings from a case control study in Sri Lanka

Supplementary table 1 - Adjusted and sex-stratified associations of parental emigration and hospital presentation for self-poisoning in adulthood (Community controls)

		n(%)			
		Cases	Controls	Model 1	Model 2
Overall	Parental migration				
	No	178 (74.5)	321 (78.3)	1	1
	Yes	61 (25.5)	89 (21.7)	1.09 (0.74, 1.60)	1.08 (0.72, 1.60)
	Which parent migrated?				
	No migration	178 (74.5)	321 (78.3)	1	1
	Maternal	36 (15.1)	55 (13.4)	1.05 (0.66, 1.68)	0.95 (0.59, 1.55)
	Paternal	21 (8.8)	30 (7.3)	1.08 (0.59, 1.97)	1.24 (0.67, 2.29)
	Both	4 (1.7)	4 (1.0)	1.65 (0.41, 6.73)	1.71 (0.41, 7.21)
Males	Parental migration				
	No	83 (79.8)	129 (77.7)	1	1
	Yes	21 (20.2)	37 (22.3)	0.76 (0.41, 1.42)	0.65 (0.34, 1.24)
Females	Parental migration				
	No	95 (70.4)	192 (78.7)	1	1
	Yes	40 (29.6)	52 (21.3)	1.36 (0.84, 2.23)	1.44 (0.87, 2.38)

Model 1 – adjusted for age and sex

Model 2 – adjusted for age, sex, ethnicity, religion, childhood SEP

Knipe D, *et al. BMJ Global Health* 2021; 6:e003734. doi: 10.1136/bmjgh-2020-003734

Supplementary material for manuscript: Is being a left-behind child associated with an increased risk of self-poisoning in adulthood? – findings from a case control study in Sri Lanka

Supplementary table 2 - Adjusted and sex-stratified associations of parental emigration and hospital presentation for self-poisoning in adulthood (All available data)

		OR 95% CI	
		Model 1	Model 2
Overall	Parental migration		
	No	1	1
	Yes	1.25 (0.87, 1.78)	1.31 (0.88, 1.93)
	Which parent migrated?		
	No migration	1	1
	Maternal	1.31 (0.85, 2.03)	1.28 (0.79, 2.09)
	Paternal	1.29 (0.72, 2.31)	1.51 (0.82, 2.79)
	Both	0.79 (0.27, 2.31)	0.83 (0.25, 2.75)
Males	Parental migration		
	No	1	1
	Yes	0.86 (0.49, 1.53)	1.57 (0.99, 2.48)
Females	Parental migration		
	No	1	1
	Yes	0.95 (0.50, 1.81)	1.70 (1.02, 2.81)

Knipe D, *et al. BMJ Global Health* 2021; 6:e003734. doi: 10.1136/bmjgh-2020-003734
